# SGLT‐2 inhibitors enhance the effect of metformin to ameliorate hormonal changes and inflammatory markers in a rat PCOS model

**DOI:** 10.14814/phy2.15858

**Published:** 2023-11-20

**Authors:** Manal Moustafa Mahmoud, Laila Ahmed Rashed, Somia Abdulatif Soliman, Safaa Mostafa Sayed, Omneya Kamel, Samaa Samir Kamar, Rania El Sayed Hussien

**Affiliations:** ^1^ Department of Physiology, Faculty of Medicine Cairo University Cairo Egypt; ^2^ Department of Biochemistry Faculty of Medicine Cairo University Cairo Egypt; ^3^ Department of Pathology, Faculty of Medicine Cairo University Cairo Egypt; ^4^ Department of Physiology, School of Medicine New Giza University Cairo Egypt; ^5^ Department of Histology, Faculty of Medicine Cairo University Cairo Egypt; ^6^ Department of Histology Armed Forces College of Medicine Cairo Egypt

**Keywords:** AMPKα‐SIRT1, empagliflozin, insulin resistance, metformin, PCOS

## Abstract

Polycystic ovary syndrome (PCOS) is a common endocrine, reproductive, and metabolic disorder affecting females. The management of PCOS is challenging and current interventions are not enough to deal with all consequences of this syndrome. We explored the beneficial effect of combined sodium glucose co transporter‐2 inhibitor (SGLT‐2i); (empagliflozin) and metformin on hormonal and metabolic parameters in an animal model of PCOS and insulin resistance (IR). Forty adult female Wistar rats divided into five groups: control, PCOS‐IR, PCOS‐IR treated with metformin, PCOS‐IR treated with empagliflozin, and PCOS‐IR treated with combined metformin and empagliflozin. Single modality treatment with metformin or empagliflozin yielded significant improvement in body mass index, insulin resistance, lipid profile, sex hormones, inflammatory markers, and ovarian cystic follicles. Combined metformin with empagliflozin expressed further significant improvement in sex hormones, inflammatory markers with disappearance of ovarian cystic follicles. The superior significant improvement with combined treatment over the single modality was in line with significant improvement in the ovarian AMPKα‐SIRT1 expression.

## Graphical abstract

Sodium glucose co transporter‐2 inhibitors (SGLT‐2i); empagliflozin combined to metformin could enhance hormonal parameters, insulin resistance, and inflammatory biomarkers in rat model of PCOS‐IR with modulating ovarian AMPK‐SIRT1 pathway which may be the molecular basis for IR in PCOS and may potentially be a therapeutic target.

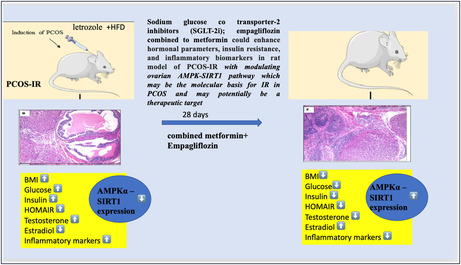

## 1 INTRODUCTION

The polycystic ovary syndrome (PCOS) is considered a common endocrine disorder in females during their reproductive age with a prevalence of 5%–10% (Tao et al., 2019). This syndrome is of unknown etiology, characterized by disturbed ovulatory function, hyperandrogenism, and polycystic ovaries. There is high prevalence of impaired glucose tolerance and insulin resistance (IR) in women with PCOS (Salley et al., 2007). The etiology of PCOS has been the subject of several theories. For instance, the hypothalamus might produce excessive amounts of gonadotropin‐releasing hormone (GnRH). The excessive luteinizing hormone (LH) that results from the GnRH surge in turn overstimulates the ovaries to make more testosterone (Rashid et al., 2022). A different PCOS syndrome explanation theory is based on endocrinological disruptions of the insulin axis, such as hyperinsulinemia and insulin resistance. Increased levels of circulating insulin reduce the liver's production of sex hormone binding globulin (SHBG). Hyperandrogenemia is brought on by the decrease in SHBG, which raises the amount of free circulating testosterone in the blood (Wallace et al., 2013). The frequency of GnRH pulses is likewise increased by hyperinsulinemia, although the LH surge predominates over the follicle stimulating hormone (FSH) surge. This causes a reduction in SHBG, an increase in ovarian androgen production, and a decrease in follicular maturation; the PCOS‐IR progresses, and eventually can lead to long‐term complications such as diabetes, fatty liver, hypertension, metabolic syndrome, ischemic heart disease, and uterine cancer (Li et al., 2019).

Accordingly, management of PCOS should not aim only to improve the symptoms, it should address both the reproductive and metabolic diseases associated with PCOS (Legro et al., 2013; Teede et al., 2018). Use of contraceptives and anti‐androgens have been the cornerstone for addressing the menstrual disturbances and hyperandrogenism (Yildiz, 2015); however, some of these treatments may fail to affect the lipid profile in PCOS and hence increase the risk of developing cardiovascular complications (Amiri et al., 2017). Weight control and lifestyle modification is one of the important components in the treatment of the disease, however, many patients fail to lose weight or quickly regain fat (Moran et al., 2011). So, effective intervention is needed to minimize these metabolic complications in women with PCOS.

Metformin and thiazolidinedione are two glucose‐lowering medications that have been used to treat PCOS' metabolic abnormalities (such as insulin resistance and diabetes mellitus) and chronic anovulation; however, their use has not consistently resulted in improvements in the management of body weight and composition, menstrual irregularities, or clinical manifestations of hyperandrogenism (Morley et al., 2017; Sam & Ehrmann, 2017).

Metformin (MF), insulin sensitizer, has been shown a treatment option that targets multiple components of PCOS (Diamanti‐Kandarakis et al., 2010). In 1994, Velazquez, reported the beneficial effects that MF had on reproductive and metabolic dysfunctions in females with PCOS. After that, many studies have confirmed the therapeutic effect of MF on IR and metabolic abnormalities in females with PCOS. MF enhances insulin sensitivity through decreasing hepatic gluconeogenesis mediated via activation of AMP‐activated protein kinase (AMPK) pathway, hence, lowers blood glucose (Kim et al., 2008). The side effects of MF are the main drawback that limit its use broadly in PCOS patients. These side effects include mainly the gastrointestinal complications such as nausea, diarrhea, and distention. In addition, the weight reducing impact of MF does not seem to be very satisfactory (Harborne et al., 2005).

Sodium glucose co transporter‐2 inhibitors (SGLT‐2i) (e.g., empagliflozin and dapagliflozin) are antidiabetic drugs that act through reducing glucose reabsorption in the proximal convoluted tubule (PCT) of the kidney via inhibition of SGLT‐2 and so increases its excretion (Chao, 2014). This class of antidiabetics has a minimal risk of hypoglycemia as it is glucose‐dependent and insulin‐independent in contrast to other antidiabetics (Scheen & Paquot, 2014). They enhance insulin sensitivity via reducing glucotoxicity and lipotoxicity, enhancement of β‐cells functions, and reducing oxidative stress and inflammation (Pereira & Eriksson, 2019). Adding to their cardiovascular and renal protective effects, SGLT2 inhibitors have been shown promising effect in controlling of the body weight (Yaribeygi et al., 2020).

Recently, in trials involving patients with PCOS, treatment with empagliflozin as opposed to metformin has demonstrated promising effects, with notable improvements in anthropometric measures and body composition. (Javed et al., 2019). This proposes the use of SGLT2 inhibitors in the management of women with PCOS. Common side effects reported for the SGLT2 inhibitors are, genitourinary tract infection and vulvovaginitis (Zinman et al., 2015). However, the exact efficacy and potential mechanism for the use of empagliflozin in PCOS has remained to be elucidated. It has been reported that the AMPK‐SIRT1 molecular pathway has a role in the pathogenesis of IR in PCOS (Tao et al., 2017). They demonstrated that that this molecular pathway may be a significant molecular contributor to IR in PCOS patients and may act as a therapeutic target for the creation of novel therapies to enhance the metabolic and reproductive health of PCOS patients.

Based on this, the aim of the present study was to investigate whether adding SGLT‐2i; empagliflozin to metformin could improve hormonal parameters, insulin resistance, and inflammatory biomarkers than metformin alone in rat model of PCOS‐IR. We aimed also to investigate whether their potential therapeutic effects were related to the AMPKα‐SIRT1 pathway. In this study, we use letrozole combined with a high fat diet for 27 days to induce a PCOS‐IR rat model which had the characteristics of ovarian polycystic changes and endocrine and metabolic disorders (Wang et al., 2020).

## 2 EXPERIMENTAL DESIGN AND TREATMENT

Forty mature female Wistar rats (weighing 160–200 g) were used in this investigation. They were acquired from Cairo University's Faculty of Medicine's Animal House. The rats were kept in wire mesh cages at room temperature with regular light and dark cycles in the animal house of Cairo University's college of medicine. This study was approved by Institutional animal care and use Committee, Cairo university. Before the study began, the animals were subjected for daily morning vaginal smear to assess the regularity of their estrous cycle. Then, the experimental rats were randomly split into five groups (eight in each).

*Group 1, (control)*: For the first 27 days, the rats in the control group were given on daily basis 0.5% carboxymethylcellulose (CMC)‐Na (1 mL/100 g/day) solution by intragastric administration along with standard diet (protein 20%, carbohydrate 70%, and fat 10%) and free water, and for the following 28 days, they were given only distilled water through orogastric gavage.

*Group 2, (PCOS‐IR)*: For 27 days, the rats in this group consumed a high fat diet (protein 20%, carbohydrate 20%, and fat 60%) along with full access to water and daily intragastric infusion of letrozole (Femara; Novartis), 1 mg/kg dissolved in 0.5% carboxymethylcellulose (CMC)‐Na (1 mL/100 g/day) (Wang et al., 2020), and for the following 28 days, they were given only distilled water through orogastric gavage.

*Group 3, (PCOS‐IR+ metformin)*: Following the induction of PCOS‐IR as in group 2 (PCOS‐IR) rats, metformin (Glucophage 500 mg; Merck Pharmaceuticals) was given via orogastric gavage at a dose of 300 mg per kilogram per day for the following 28 days. The amount of metformin utilized in this trial was comparable to the dosage given to PCOS patients (Reagan‐Shaw et al., 2008; Sander et al., 2006).

*Group 4, (PCOS‐IR+ empagliflozin)*: Following the induction of PCOS‐IR as in group 2 (PCOS‐IR) rats, empagliflozin (EMPA, 10 mg/kg/day) was administered via orogastric gavage (Woods et al., 2019) for the following 28 days.

*Group 5, (PCOS‐IR+ combined metformin + empagliflozin)*: Following the induction of PCOS‐IR as in group 2 (PCOS‐IR) rats, then were administered metformin (Glucophage 500 mg; Merck Pharmaceuticals) at 300 mg / kg /day and empagliflozin (EMPA, 10 mg/kg/day) via orogastric gavage for the next 28 days.

### 2.1 Experimental measurements

- The body weights and nose‐anus lengths were measured weekly, and BMI were calculated according to the formula (Novelli et al., 2007):

Body mass index (BMI) = body weight (g)/square length (cm^2^)

- Vaginal epithelial cell smears were performed in all experimental rats after 4 weeks and 8 weeks from the start of the experiment to assess the stages of the rats' estrous cycle (Salama et al., 2020).
- Blood samples were taken through the retro‐orbital plexus at the conclusion of the 8‐week research period and centrifuged for 20 min at 12298 g. The serum was then isolated and kept at −70°C for use in estimating the parameters listed below:

1. Fasting serum glucose, insulin, and insulin resistance (HOMA‐IR): Using tools provided by “Diamond Diagnostics,” (Holliston, MA), the serum glucose level was tested as previously described (Trinder, 1969). Rat insulin ELISA kits were used to perform an enzyme immunoassay to measure the serum insulin concentrations. (Gallois et al., 1996), and HOMA‐IR index was then calculated with the following equation: (Emoto et al., 1999).
$$\begin{array}{c} \frac{\text{Fasting plasma insulin}\;\left(\mathrm{FPI}\right)\;\left(\mathrm{mIU}\right)\times \text{fasting plasma glucose}\;\left(\mathrm{FPG}\right)\;\left(\text{mmol}/L\right)}{22.5} \end{array}$$
2. Lipid profile (Total cholesterol, Triglycerides, and HDL): Total serum cholesterol, HDL, and triglyceride were measured using the conventional quantitative enzymatic colorimetric method (Allain et al., 1974), (Buckley et al., 1966).
3. Hormonal assay: Serum hormone levels were assessed as previously mentioned (Zhao et al., 2017). Using ELISA Kits, the levels of serum FSH, LH, testosterone, and estradiol were determined (Enzo Life Science, Inc., Farmingdale, NY, USA).The procedure was followed as given in the kit catalog (Stat Fax® 2100, Fisher Bioblock Scientific, France).
4. Serum pro‐inflammatory cytokines: Measurement of serum tumor necrosis factor α; TNF α was measured via quantitative sandwich enzyme immunoassay technique (Kinra & Dutta, 2013). The serum interleukin‐6; IL6 was measured by using the ELISA (Quantikine; R&D Systems) following the manufacturer's instructions (Hirano, 1998).

#### 2.1.1 Tissue samples

Rats were sacrificed by decapitation at the conclusion of the 8‐week research period, and their ovaries were dissected and cleansed of fat. The left ovaries were immediately fixed in 10% buffered formalin, embedded in paraffin wax then processed as paraffin blocks. The right ovaries were rinsed in ice‐cold saline, divided into small pieces, and stored at 80°C until employed for biochemical determination of relative gene expression and protein levels of AMPK and SIRT1 by real‐time PCR and western blot following the protocols below:

- Total RNA extraction: RNeasy system was used to further purify the total RNA after it had been obtained using Trizol reagent (Invitrogen, Carlsbad, CA) (Qiagen, Valencia, CA). A UV spectrophotometer was used to measure the quantities and purity of RNA.
- Complementary DNA (cDNA) synthesis: Using the Super Script III First‐Strand Synthesis System and the manufacturer's instructions, 1 g of RNA was converted into cDNA (#K1621, Fermentas, Waltham, MA, USA).
- Real‐time quantitative PCR Real‐time: Using an Applied Biosystem with software version 3.1, PCR amplification and analysis were carried out (Step One™, USA). Using RNA sequences from the gene bank, Gene Runner Software (Hasting Software, Inc., Hasting, NY) was used to make gene‐specific primer pairs. The SYBR Green Master Mix was used in the reaction (Applied Biosystems). Each primer set's annealing temperature was calculated to be 60 degrees. In a 25 μL reaction volume, quantitative RT‐PCR was carried out using 2X SYBR Green PCR Master Mix (Applied Biosystems), 900 nM of each primer, and 2 μL of cDNA.

*Amplification conditions were*: 2 min at 50°, 10 min at 95°, and 40 cycles of 15‐second denaturation followed by 10 min of annealing/extension at 60°. Using PE Biosystems' v1‐7 sequence detection software, real‐time assay data were calculated (Foster City, CA). The comparative Ct technique was used to determine the relative expression of the investigated gene's mRNA. Beta actin was used as the control housekeeping gene and all values were normalized against it and reported as fold change over background levels detected in the diseased groups.

*The primer sequence of the AMPKα gene*:

Forward Primer: 5′‐TAAACC CACAGAAATCCAAACACC‐3′

Reverse primer 5′‐ACAACC TTCCATTCATAGTCCAACT‐3′

*The primer sequence of the SIRT1 gene*:

Forward Primer: 5′‐AACCACCAAAGCGGAAAAAAAGAA‐3′

Reverse primer 5′‐CCACAGCAAGGCGAGCATAAATA‐3′

*Specific primer sequence for b‐actin (used as housekeeping gene)*:

Forward Primer: 5′‐CCGTAAAGACCTCT ATGCCAACA‐3′

Reverse primer 5′‐CTAGGAGCCAGGGC AGTAATCTC‐3′

#### 2.1.2 Histological study

Paraffin blocks were sectioned using a microtome at a thickness of 5 microns. The ovarian slides stained with hematoxylin & eosin (H&E) were blindly examined by two MD‐investigators using light microscopy connected to digital camera (Olympus, Japan) to assess the ovarian follicles. Five non‐overlapping fields/ H&E section/ rat, at magnification ×100, were evaluated for counting the sum of Graafian follicles, displaying a large fluid‐filled antral cavity surrounded by granulosa cells and containing oocyte, and the corpora leutea.

### 2.2 Western blot for AMPK and SIRT1 levels

For Western blot analysis, tissues were homogenized in the homogenization buffer containing 50 mM Tris pH 7.4, 10 mM NaF, 2 mM EDTA, 10 mM β‐glycerol phosphate, 1 mM Na3VO4, 0.2% W/V sodium deoxycholate, 1 mM phenylmethylsulfonyl fluoride (PMSF), and complete protease inhibitor cocktail (Sigma, P8340) employing polytron homogenizer (POLYTRON_ PT 10–35, Kinematica, Switzerland) in ice. The lysates were centrifuged at 10,000 g at 4°C for 15 min. The Bradford protein assay kit (Bio‐Rad) was used to measure the protein concentration in the supernatants. AMPK, SIRT1, and actin levels were assessed by immunological blotting analysis. Then, the separated samples were transferred to a polyvinylidene fluoride (PVDF) membrane. To summarize, the produced samples were combined with loading buffer and heated for 8 min at 95°C. The blots were incubated for 2 h at room temperature in blocking buffer TBS‐T (5% nonfat milk and 0.1% Tween‐20 in Tris‐buffered saline). The primary antibodies for AMPK, Sirt1, and beta actin were provided by Thermo Fisher and utilized at a dilution of 1:000. After being exposed to the primary antibodies for an overnight period at 4°C, the blots were washed three times for 10 min each in TSB containing 0.1% Tween‐20. The membranes were then cleaned as previously mentioned and treated with the matching secondary antibody. Alliance gel doc (Alliance 4.7 Gel doc, UK) and enhanced chemiluminescence (Pierce ECL Western blotting substrate) were used to see protein bands. Protein bands were semi‐quantified using UV Tec software (UK). The beta actin protein was used to normalize all protein bands.

### 2.3 Statistical methods

The data were coded and entered using SPSS version 28 (Statistical Package for the Social Sciences) (IBM Corp., Armonk, NY, USA). Results were presented as mean ± standard deviation (SD) using one‐way analysis of variance (ANOVA) followed by Bonferroni post‐hoc multiple comparison test.

## 3 RESULTS

### 3.1 Confirmation of PCOS

The cytology of the vaginal swab was examined to confirm the induction of PCOS. Epithelial cells with a small number of keratinocytes are present during the proestrus stage; keratinocytes are present during the estrus; keratinocytes, leukocytes, and epithelial cells are present during the metestrus; and predominantly leucocytes are present during the diestrus. In a PCOS‐IR model, BMI is considerably improved by metformin, empagliflozin, and their combination.

As shown in Table 1, significant increase in BMI was observed in PCOS‐IR rats as compared with control group (0.83 ± 0.09 vs. 0.57 ± 0.06; *p* < 0.05). Metformin treatment resulted in significant improvement in the elevated BMI (0.63 ± 0.05 vs. 0.83 ± 0.09; *p* < 0.05). Empagliflozin treatment also showed a similar effect on PCOS‐IR rats to that of metformin on BMI (0.64 ± 0.07 vs. 0.83 ± 0.09; *p* < 0.05). Combined metformin with empagliflozin expressed significant decrease in the elevated BMI values (0.55 ± 0.04 vs. 0.57 ± 0.06; *p* < 0.05), however, it did not show significant superior effect to single modality treatment.

**TABLE 1:** Comparison of the mean value of BMI, serum glucose, serum insulin, HOMA‐IR, total cholesterol, total triglycerides, HDL, LDL, FSH, LH, LH/FSH ratio, testosterone, estradiol, TNF‐ α, IL6 of the PCOS‐IR rat model subjected for 28 days of empagliflozin, metformin monotreatment, and combined drug treatment. One‐way ANOVA was used as a statistical test.

	Control	PCOS‐IR	PCOS‐IR+ metformin	PCOS‐IR+ empagliflozin	PCOS‐IR+ combined metformin+ empagliflozin
BMI	0.57 ± 0.06	0.83 ± 0.09*	0.63 ± 0.05^#^	0.64 ± 0.07^#^	0.55 ± 0.04^#^
serum glucose mmol/mL	5.23 ± 0.64	16.4 ± 1.81*	8.3 ± 0.92*, ^#^	8.08 ± 0.67* ^,^ ^#^	5.93 ± 0.88^#^ ^,^ ^$^ ^,^ ^@^
serum insulin μIU	9.01 ± 0.85	20.74 ± 1.75*	11.8 ± 0.69* ^,^ ^#^	13.31 ± 1.26* ^,#^	10.33 ± 1.16^#^ ^,^ ^@^
HOMA‐IR	2.07 ± 0.36	15.14 ± 2.39*	4.3 ± 0.63* ^,^ ^#^	4.74 ± 0.71* ^,#^	2.69 ± 0.51^#^ ^,^ ^@^
Total cholesterol mg/dL	143.63 ± 7.21	256.13 ± 14.12*	178.63 ± 9.33* ^,^ ^#^	186.38 ± 13.57* ^,^ ^#^	187.13 ± 8.71* ^,^ ^#^
Triglycerides mg/dL	81.88 ± 9.69	134.5 ± 11.86*	94.38 ± 8.63^#^	94.88 ± 9.63^#^	79 ± 8.38^#^ ^,^ ^$^ ^,^ ^@^
HDL mg/dL	58.25 ± 5.99	31.37 ± 6.55*	48.75 ± 6.27* ^,^ ^#^	48.88 ± 6.27* ^,^ ^#^	52.38 ± 5.07^#^
LDL mg/dL	68.57 ± 8.74	197.85 ± 12.26*	111 ± 8.82* ^,^ ^#^	118.53 ± 14.19* ^,^ ^#^	118.95 ± 6.29* ^,^ ^#^
FSH mIU/mL	1.49 ± 0.37	5.54 ± 0.83*	2.1 ± 0.23^#^	2 ± 0.31^#^	1.52 ± 0.38^#^
LH mIU/mL	13.48 ± 2.27	53.54 ± 7.81*	22.14 ± 2.23* ^,^ ^#^	22.56 ± 2.33* ^,^ ^#^	16.1 ± 2.1^#^ ^,^ ^$^ ^,^ ^@^
LH/FSH ratio	9.35 ± 1.93	9.97 ± 2.75	10.6 ± 1.62	11.47 ± 2.1	11.01 ± 2.31
Testosterone ng/mL	1.59 ± 0.35	8.27 ± 0.59*	4.11 ± 0.91* ^,^ ^#^	4.14 ± 0.58* ^,^ ^#^	1.93 ± 0.22^#^ ^,^ ^$^ ^,^ ^@^
Estradiol pg/mL	128.73 ± 9.35	63.1 ± 6.82*	110.56 ± 3.05* ^,^ ^#^	109.96 ± 3.62* ^,^ ^#^	122.85 ± 3.68^#^ ^,^ ^$^ ^,^ ^@^
TNF‐α (pg/mL)	32.03 ± 3.63	133.84 ± 31.5*	76.21 ± 8.47* ^,^ ^#^	76.02 ± 6.17* ^,^ ^#^	50.41 ± 7.07^#^ ^,^ ^$^ ^,^ ^@^
IL6 pg/mL	31.23 ± 3.52	127.21 ± 5.63*	70.36 ± 5.86* ^,^ ^#^	68.78 ± 8.44* ^,^ ^#^	46.64 ± 4.85* ^,^ ^#^ ^,^ ^$^ ^,^ ^@^

*Note*: Values are presented as mean ± SD.

* Statistically significant compared to corresponding value in control group (*p* < 0.05).

^#^ Statistically significant compared to corresponding value in PCOS‐IR group (*p* < 0.05).

^$^ Statistically significant compared to corresponding value in PCOS‐IR+ metformin group (*p* < 0.05).

^@^ Statistically significant compared to corresponding value in PCOS‐IR+ empagliflozin group (*p* < 0.05).

### 3.2 Metformin, empagliflozin, and their combination ameliorate insulin resistance in a PCOS‐IR model

Insulin resistance is one of the basic metabolic abnormalities observed in obese patients with PCOS. As shown in Table 1, in PCOS‐IR rats, the fasting insulin was higher than that in the control rats (20.74 ± 1.75 vs. 9.01 ± 0.85; *p* < 0.05), Fasting glucose levels also manifested significant increase in PCOS‐IR rats compared with control rats (16.4 ± 1.81 vs. 5.23 ± 0.64; *p* < 0.05). HOMA‐IR increased significantly in PCOS‐IR rats compared with control rats (15.14 ± 2.39 vs. 2.07 ± 0.36; *p* < 0.05), suggesting insulin resistance in PCOS‐IR rats' model. Metformin treatment resulted in significant decrease in the elevated insulin, glucose levels, and the HOMA‐IR in PCOS‐IR rats (fasting insulin, 11.8 ± 0.69; glucose, 8.3 ± 0.92; HOMA‐IR, 34.3 ± 0.63; *p* < 0.05). Empagliflozin treatment showed a similar effect on PCOS‐IR rats to that of metformin (fasting insulin, 13.31 ± 1.26; glucose, 8.08 ± 0.67; HOMA‐IR, 4.74 ± 0.71; *p* < 0.05). Furthermore, combined metformin with empagliflozin significantly enhanced the inhibitory effect of the respective empagliflozin alone on the insulin, glucose levels, and HOMA‐IR in PCOS‐IR rats (fasting insulin, 10.33 ± 1.16; glucose, 5.93 ± 0.88; HOMA‐IR, 2.69 ± 0.51; *p* < 0.05). However, combined metformin with empagliflozin did not significantly enhance the inhibitory effect of the respective metformin alone on the insulin levels and HOMA‐IR in PCOS‐IR rats with only significant decrease in the glucose levels (5.93 ± 0.88; *p* < 0.05). The results showed that metformin and empagliflozin combination therapy reduced the high levels of insulin and ameliorated insulin resistance in PCOS‐IR as compared with empagliflozin alone.

### 3.3 Metformin, empagliflozin and their combination ameliorate serum lipid profile in a PCOS‐IR model

Disturbed lipid profile is an important aspect in PCOS‐IR patients. As shown in Table 1, in PCOS‐IR rats, total cholesterol was significantly higher than that in the control rats (256.13 ± 14.12 vs. 143.63 ± 7.21; *p* < 0.05), triglycerides level also showed significant increase in PCOS‐IR rats compared with control rats (134.5 ± 11.86 vs. 81.88 ± 9.69; *p* < 0.05). Serum HDL decreased significantly in PCOS‐IR rats compared with control rats (31.37 ± 6.55 vs. 58.25 ± 5.99; *p* < 0.05).Use of metformin, empagliflozin, and their combination showed significant improvement in total cholesterol level (PCOS‐IR Met, 178.63 ± 9.33; PCOS‐IR Empa, 186.38 ± 13.57; PCOS‐IR Met+Empa, 187.13 ± 8.71 vs. PCOS‐IR 256.13 ± 14.12 *p* < 0.05), Triglycerides level (PCOS‐IR Met, 94.38 ± 8.63; PCOS‐IR Empa, 94.88 ± 9.63; PCOS‐IR Met+Empa, 79 ± 8.38 vs. PCOS‐IR 134.5 ± 11.86 *p* < 0.05), and HDL level (PCOS‐IR Met, 48.75 ± 6.27; PCOS‐IR Empa, 48.88 ± 6.27; PCOS‐IR Met+Empa, 52.38 ± 5.07 vs. PCOS‐IR 31.37 ± 6.55 *p* < 0.05). Combined metformin with empagliflozin significantly enhanced the effect of the respective empagliflozin alone on triglycerides, otherwise, no significant difference observed for combined metformin with empagliflozin over single modality treatment on lipid profile.

### 3.4 Metformin, empagliflozin, and their combination ameliorate sex hormone disturbances in a PCOS‐IR model

Hyperandrogenism is the corner stone in PCOS and insulin resistant patients. As shown in Table 1, in PCOS‐IR rats, the testosterone level was higher than that in the control rats (8.27 ± 0.59 vs. 1.59 ± 0.35; *p* < 0.05), estradiol levels also showed significant decrease in PCOS‐IR rats compared with control rats (63.1 ± 6.82 vs. 128.73 ± 9.35; *p* < 0.05). LH level decreased significantly in PCOS‐IR rats compared with control rats (53.54 ± 7.81 vs. 13.48 ± 2.27; *p* < 0.05), while FSH showed significant increase in PCOS‐IR rats compared with control rats (5.54 ± 0.83 vs. 1.49 ± 0.37; *p* < 0.05) indicating characteristic hormonal disturbances in PCOS‐IR rats model. Metformin or empagliflozin treatment of PCOS‐IR rats significantly decreased the level of testosterone (PCOS‐IR Met, 4.11 ± 0.91; PCOS‐IR Empa, 4.14 ± 0.58; vs. PCOS‐IR, 8.27 ± 0.59 *p* < 0.05). Furthermore, use of metformin or empagliflozin showed significant improvement in estradiol level (PCOS‐IR Met, 110.56 ± 3.05; PCOS‐IR Empa, 109.96 ± 3.62; vs. PCOS‐IR 63.1 ± 6.82 *p* < 0.05), LH levels (PCOS‐IR Met, 22.14 ± 2.23; PCOS‐IR Empa, 22.56 ± 2.33; vs. PCOS‐IR 53.54 ± 7.81 *p* < 0.05), and FSH levels (PCOS‐IR Met, 2.1 ± 0.23; PCOS‐IR Empa, 2 ± 0.31; vs. PCOS‐IR 5.54 ± 0.83 *p* < 0.05). The improvement of sex hormones was intensified with combined treatment of metformin and empagliflozin compared to that with single treatment (testosterone, 1.93 ± 0.22; estradiol, 122.85 ± 3.68; LH, 16.1 ± 2.1; FSH, 1.52 ± 0.38 *p* < 0.05).

### 3.5 Polycystic ovary and insulin resistance significantly increased the levels of inflammatory markers; TNF‐α and IL6, which was reversed by intake of metformin, empagliflozin, and their combination

Inflammation is one of the most potent risk factors of PCOS. As shown in Table 1, there was a significant increase in the mean values of TNF‐α and IL6 in PCOS‐IR rats than that in the control rats (133.84 ± 31.5 and 127.21 ± 5.63 vs. 32.03 ± 3.63 and 31.23 ± 3.52; *p* < 0.05 respectively). Use of metformin, empagliflozin, and their combination showed significant improvement in TNF‐α levels (PCOS‐IR Met, 76.21 ± 8.47; PCOS‐IR Empa, 76.02 ± 6.17; PCOS‐IR Met+Empa, 50.41 ± 7.07 vs. PCOS‐IR 133.84 ± 31.5 *p* < 0.05), and IL6 levels (PCOS‐IR Met, 70.36 ± 5.86; PCOS‐IR Empa, 68.78 ± 8.44; PCOS‐IR Met+Empa, 46.64 ± 4.85 vs. PCOS‐IR 127.21 ± 5.63 *p* < 0.05). Interestingly, significant improvement of the inflammatory markers was noticed with combined treatment of metformin and empagliflozin compared to single modality treatment.

### 3.6 Metformin, empagliflozin, and their combination improve ovarian cystic follicles and metabolic disturbances in PCOS‐IR model through the AMPKα‐SIRT1 pathway

**FIGURE 1:**
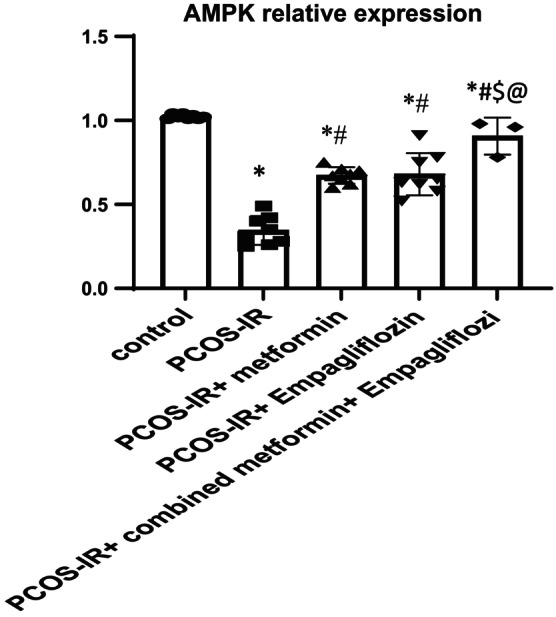
Comparison of the mean value of AMPKα gene expression (PCR of the PCOS‐IR rat model subjected for 28 days of empagliflozin, metformin monotreatment, and combined drug treatment. One‐way ANOVA was used as a statistical test. Values are presented as mean ± SD. *: statistically significant compared to corresponding value in control group (*p* < 0.05). #: statistically significant compared to corresponding value in PCOS‐IR group (*p* < 0.05). $: statistically significant compared to corresponding value in PCOS‐IR+ metformin group (*p* < 0.05). @: statistically significant compared to corresponding value in PCOS‐IR+ empagliflozin group (*p* < 0.05).

**FIGURE 2:**
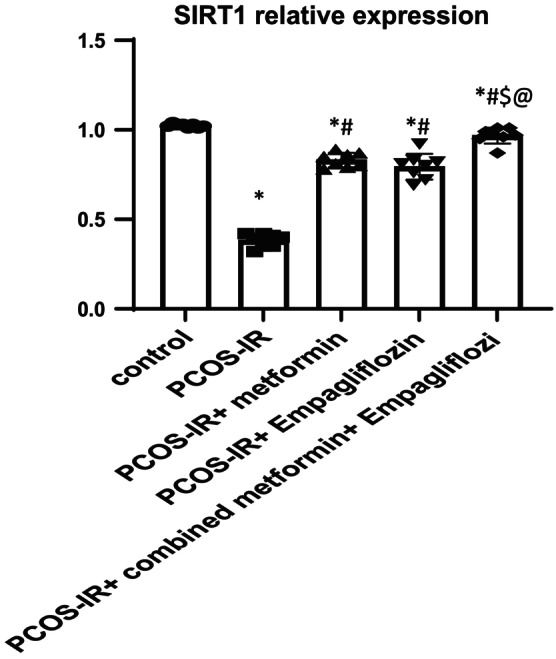
Comparison of the mean value of SIRT 1 gene expression of the PCOS‐IR rat model subjected for 28 days of empagliflozin, metformin monotreatment, and combined drug treatment. One‐way ANOVA was used as a statistical test. Values are presented as mean ± SD. *: statistically significant compared to corresponding value in control group (*p* < 0.05). #: statistically significant compared to corresponding value in PCOS‐IR group (*p* < 0.05). $: statistically significant compared to corresponding value in PCOS‐IR+ metformin group (*p* < 0.05). @: statistically significant compared to corresponding value in PCOS‐IR+ empagliflozin group (*p* < 0.05).

**FIGURE 3:**
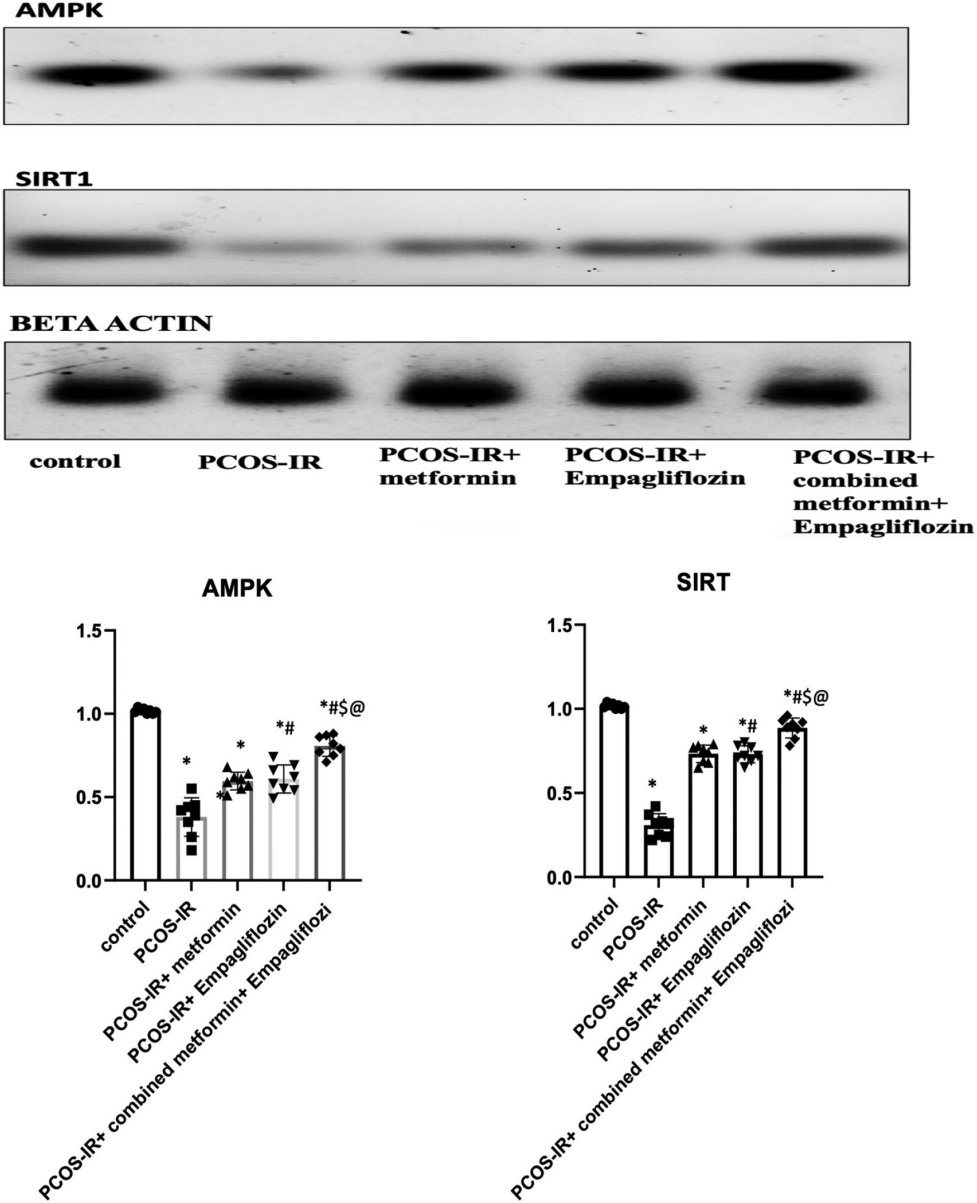
Expression of AMPK and SIRT1 in the ovarian tissue homogenate of the PCOS‐IR rat model subjected for 28 days of empagliflozin, metformin monotreatment, and combined drug treatment shown in the upper panel. Beta actin confirms equal loading of the protein. Western blot was performed in triplicates with similar results. One representative blot is shown. One‐way ANOVA was used as a statistical test. Values are presented as mean ± SD. *: statistically significant compared to corresponding value in control group (*p* < 0.05). #: statistically significant compared to corresponding value in PCOS‐IR group (*p* < 0.05). $: statistically significant compared to corresponding value in PCOS‐IR+ metformin group (*p* < 0.05). @: statistically significant compared to corresponding value in PCOS‐IR+ empagliflozin group (*p* < 0.05).

As shown in Figure 1, there was a significant decrease in the relative expression of AMPKα in ovarian tissue in PCOS‐IR rats than that in the control rats (0.38 ± 0.12 vs. 1.02 ± 0.01; *p* < 0.05). Use of metformin, empagliflozin, and their combination showed significant improvement in the relative expression of AMPKα in ovarian tissue (PCOS‐IR Met, 0.6 ± 0.05; PCOS‐IR Empa, 0.61 ± 0.08; PCOS‐IR Met+Empa, 0.81 ± 0.06 vs. PCOS‐IR 0.38 ± 0.12 ± 31.5 *p* < 0.05). Also, there was a significant decrease in the relative expression of SIRT1 in ovarian tissue in PCOS‐IR rats than that in the control rats (0.31 ± 0.07 ± 5.63 vs. 11.02 ± 0.01; *p* < 0.05) (Figure 2).We reported significant improvement in the relative expression of SIRT1 levels with use of metformin, empagliflozin, and their combination (PCOS‐IR Met, 0.73 ± 0.05; PCOS‐IR Empa, 0.73 ± 0.05; PCOS‐IR Met+Empa, 0.89 ± 0.06 vs. PCOS‐IR 0.38 ± 0.12 ± 31.5 *p* < 0.05). We noticed that use of combined treatment of metformin and empagliflozin significantly improved the relative expression of AMPKα and SIRT1 in PCOS‐IR rats compared to single modality treatment. In Figure 3, we also noticed with western blot analysis of in ovarian homogenate that protein levels of AMPKα and SIRT1 were improved significantly with combined metformin and empagliflozin treatment.

### 3.7 Metformin, empagliflozin, and their combination restore the regularity of estrous cycle and improve the maturation of ovarian follicles in the PCOS‐IR model

**FIGURE 4:**
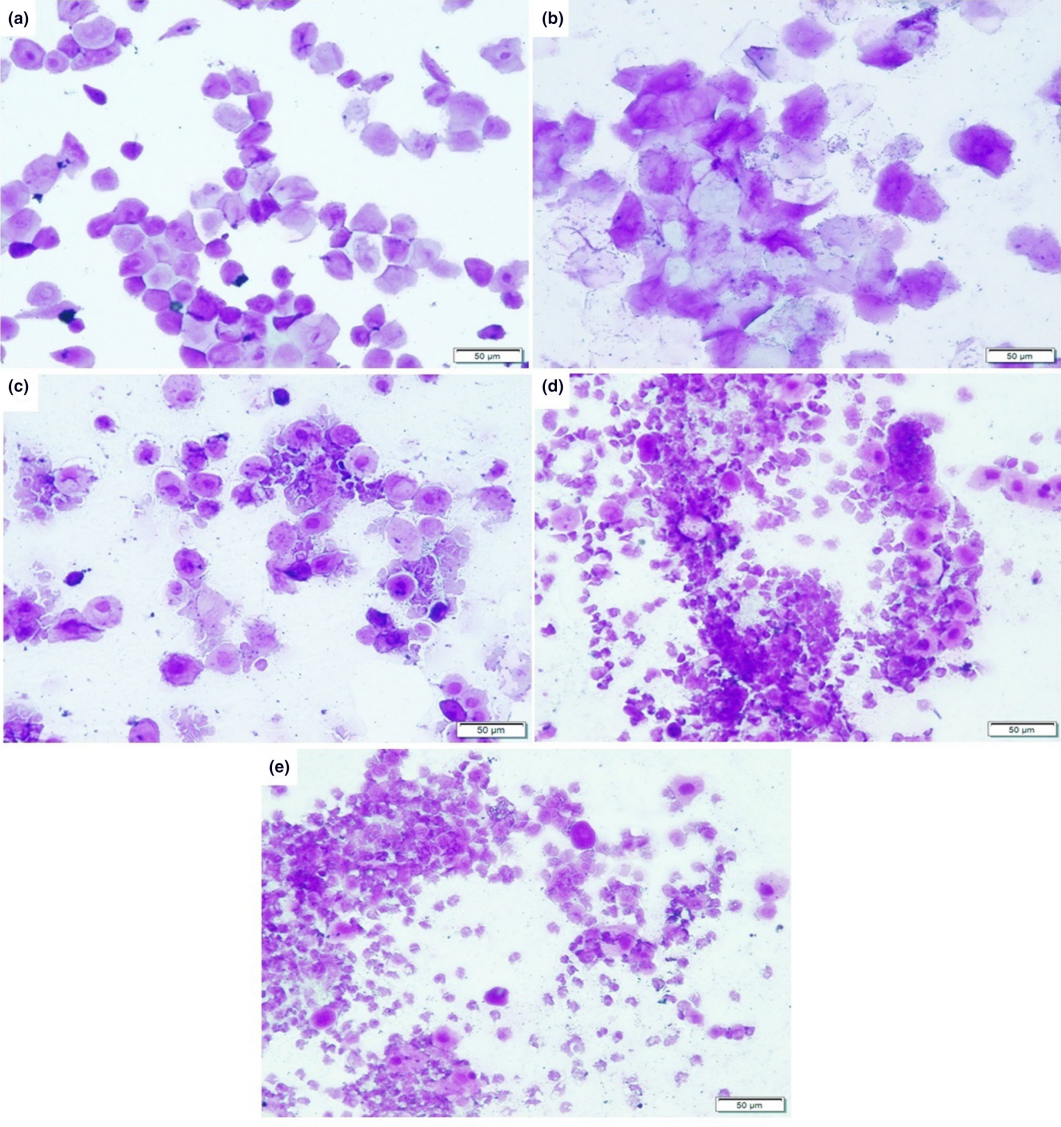
Vaginal epithelial cell smears stained with crystal violet for the control (a–d) and PCOS‐IR rat model. (a) Proestrus phase; (b) estrus phase; (c) metaestrus phase; and (d) diestrus phase. (e) Numerous leukocytes with scanty nucleated epithelium. (Magnification: ×200).

All rats showed regular estrous cycle all through 4 days of examination before the beginning of the experiment (data not shown). Besides, the control rats showed regular estrous cycle after 4 and 8 weeks of the start of the experiment; proestrus, estrus, metestrus, and diestrous (Figures 4a–d). All rats of PCOS rats illustrated prolonged diestrus phase after 4 and 8 weeks (Figures 4e). On single modality treatment, five rats with each treatment experienced epithelial keratinocytes observed microscopically during the vaginal smears at the end of the experiment. However, seven rats with the combined treatment displaced regular phases of the estrous cycle in the vaginal smears at the end of the experiment that was comparable to the control rats (data not shown).

**FIGURE 5:**
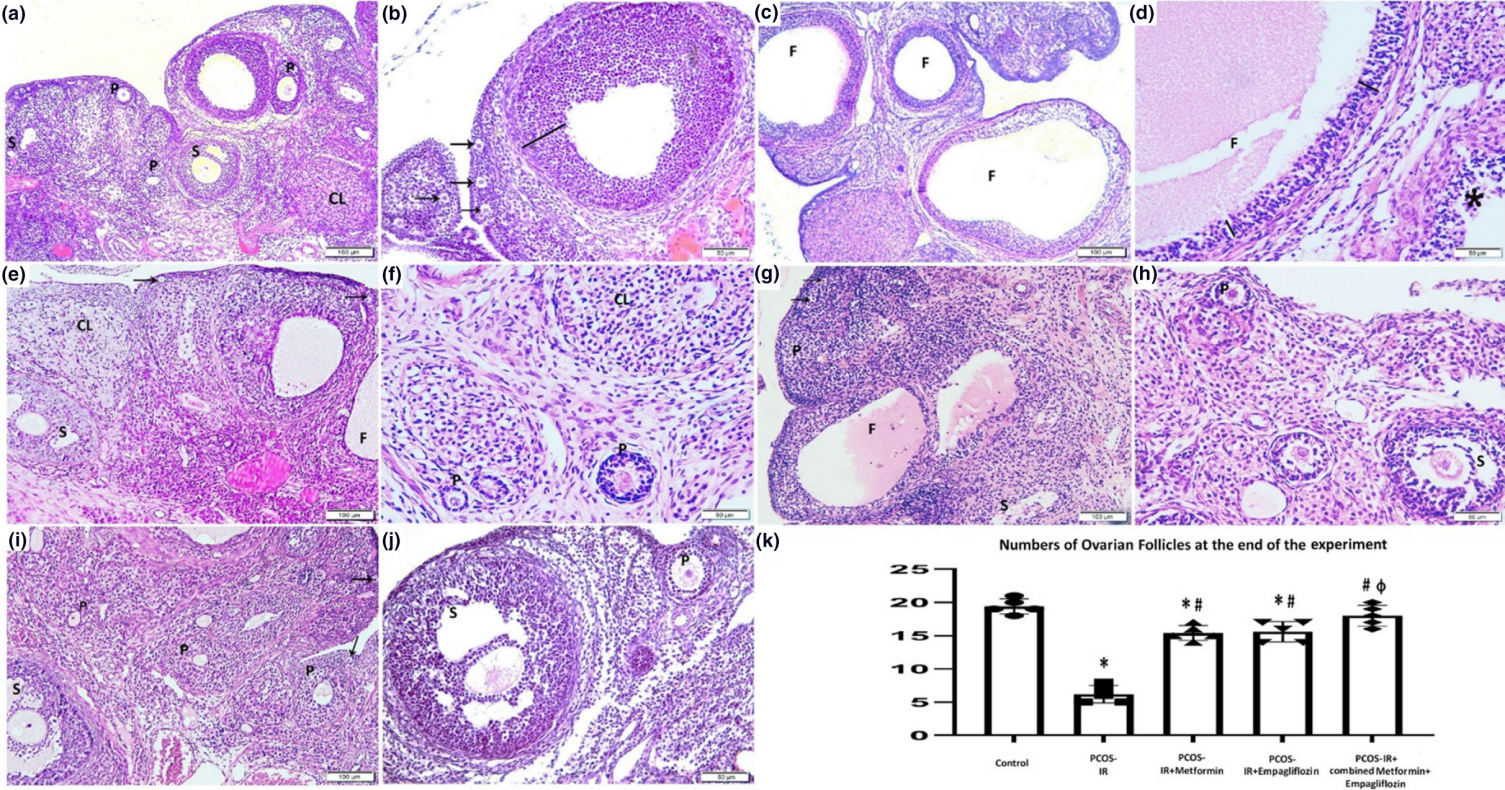
Photomicrographs of H&E sections in rat ovarian tissue of the PCOS‐IR rat model subjected for 28 days of empagliflozin, metformin monotreatment, and combined drug treatment. (a, b) Control group: multiple cortical follicles in different stages of maturation illustrating thick granuolosa cell layers (line). (c, d) PCOS‐IR group: multiple enlarged cystic follicles (f) compressing the ovarian tissue and displaying thin‐walled granulosa cell layer (line). Disrupted granulosa cells are noted (asterisk). (e, f) PCOS‐IR + metformin: reduction in the size of the cystic follicles with increased thickness of granulosa cell layer. (g, h) PCOS‐IR + empagliflozin: reduction in the size of cystic follicles with more increase in thickness of granulosa cell layer. (i, j) PCOS‐IR + combined metformin+empagliflozin: apparently normal growing follicles at different stages of maturation with disappearance of cystic follicles. (Arrow: primordial follicle; P: primary follicle; S: secondary follicle; CL: corpus leuteum) (Magnifications: a, c, e, g, i ×100; b, d, f, h, j ×200). (K) The number of the ovarian follicles in the control and the experimental groups. Data was presented as mean (± SD) using one‐way ANOVA test. * Significant (*p* < 0.05) as compared to the control group; # significant as compared to PCOS‐IR group; ɸ significant as compared to PCOS‐IR + metformin and PCOS‐IR + empagliflozin groups.

Normal ovarian follicles at various stages of maturity were visible in control ovarian rat models. There were many cystic follicles with thin granulosa cell layer and compressed ovarian stroma in PCOS‐IR rats with a significant decrease in the number of mature Graafian follicles and corpora leutea. The size and number of the ovarian cystic follicles were reduced with the use of metformin or empagliflozin, and the thickness of the granulosa cell layer was increased. Meanwhile, using metformin and empagliflozin as a combined treatment led to restoration of the follicular growth and maturation into different stages with significant increase in the number of the mature ovarian follicles and corpora leutea as compared to other experimental groups. (Figure 5).

## 4 DISCUSSION

Polycystic ovarian syndrome (PCOS) is a complex endocrine disorder that affects women with high prevalence of cardiometabolic risk factors, such as type 2 diabetes mellitus (T2DM), insulin resistance, metabolic syndrome, cardiovascular disease, and higher incidence of endometrial cancer (Burt Solorzano & McCartney, 2021; Yumiceba et al., 2020). Despite this, there are currently a dearth of efficient, evidence‐based treatments for such hormonal and metabolic disorders. In the present study, letrozole combined with a high fat diet for 27 days exhibited features of PCOS‐IR including ovarian cystic changes, sex hormone disturbances, insulin resistance together with weight gain, dyslipidemia, and elevated inflammatory markers.

One of the main traits of PCOS is IR, with compensatory hyperinsulinemia. Both IR and hyperinsulinemia are linked to metabolic syndrome and can lead to abnormalities in reproduction. There is proof that insulin sensitizers like metformin can enhance PCOS patients' reproductive health. (Fulghesu et al., 2012; Pasquali, 2015).

Dyslipidemia is one of the rather common metabolic issues in women with PCOS. Since insulin resistance is a primary pathophysiology of PCOS, dyslipidemia in PCOS‐afflicted women may be consistent with cases of the condition that have been documented in an insulin resistant state. In the present study, we demonstrated that empagliflozin, metformin monotreatment, and combined drug treatment of PCOS‐IR rat model for 28 days exhibited significant improvement in HOMA‐IR and dyslipidemia where combined metformin and empagliflozin has shown significant superior effect to empagliflozin but not to metformin monotreatment.

Accumulating evidence have examined how insulin resistance in PCOS women could be managed by metformin, empagliflozin, either alone or in combination with other treatments. (Abuelghar et al., 2013; El Maghraby et al., 2015; El‐khayat et al., 2016; Mhao et al., 2016).In accordance with our findings, study done on efficacy of canagliflozin versus metformin in women with PCOS revealed that both drugs can significantly improve HOMA‐IR in women having PCOS with IR without significant difference between them (Cai et al., 2022). Several lines of evidence point to beneficial effect of metformin in improving HOMA‐IR in PCOS women (Li et al., 2020; Tan et al., 2008). Proposed mechanisms of action of metformin are through minimizing hepatic glucose output and improving the insulin‐mediated uptake of glucose (Abdalla et al., 2020).

SGLT2 inhibitors also known to enhance insulin sensitivity via multiple pathways as facilitating β‐cells function, caloric deposition and reducing glucotoxicity, lipotoxicity, oxidative damage, and inflammatory processes (Yaribeygi et al., 2020).

Numerous studies have demonstrated that metformin has a considerable impact on dyslipidemia (Fleming et al., 2002; Ng et al., 2001), either directly by affecting how the liver processes free fatty acids or indirectly by lowering hyperinsulinemia (Sin et al., 2011).

SGLT‐2 inhibitors also affect plasma lipids as studies have been shown that use of SGLT‐2 inhibitors results in a reduction in triglycerides, increases in HDL and LDL‐cholesterol levels (Briand et al., 2016; Inzucchi et al., 2015). It has been hypothesized that hemoconcentration happened upon use of SGLT2 inhibitors as a result of increased urinary volume could be the contributing factor to the reported high LDL‐C levels (Lund et al., 2016; Pieber et al., 2015).

In contrast to our results, Javed et al., 2019 performed RCT, comparing hormonal and metabolic effect of empagliflozin versus metformin in PCOS women; where they did not report significant changes in fasting glucose, insulin, or lipids after 3 months of treatment. They explained their findings by pointing to the brief study duration or the youthful age and lack of diabetes of their PCOS participants.

Insulin resistance (IR) is tissue‐selective in PCOS patients, as evidenced by the fact that the ovaries and adrenal glands continue to respond to insulin despite skeletal muscles, adipose tissue, and the liver losing their sensitivity to it (Dabadghao, 2019). By stimulating insulin's receptors in the follicle membrane cells, which in turn triggers androgen synthesis in ovarian theca cells, insulin successfully promotes ovarian follicle development and hormone release. Additionally, through its receptors in the pituitary gland, hyperinsulinemia causes the release of LH (Rothenberg et al., 2018).

Study on PCOS women has shown that high testosterone levels are capable of being converted to estrone in adipose tissue. Increased conversion of estrone to estradiol, which alters follicle development and increases the LH to FSH ratio, causes ovulatory dysfunction. (Li et al., 2019).

Hyperandrogenism (HA) raised the LH levels as a result of disturbed the negative feedback effect of estradiol and progesterone on GnRH (Ibáñez et al., 2017). Additionally, HA worsens IR through a variety of mechanisms, including decreased insulin sensitivity, decreased GLUT‐4 expression, and decreased insulin breakdown in the liver. A kind of skeletal muscle fiber with low insulin sensitivity is also increased by HA (Wang et al., 2019). On the other hand, HA exacerbates central obesity, a factor in IR (Li et al., 2019). In the present study, we demonstrated that metformin or empagliflozin treatment of PCOS‐IR rats significantly decreased the level of testosterone and induced significant improvement in estradiol, LH, and FSH levels. The improvement of sex hormones was intensified with combined treatment of metformin and empagliflozin compared to that with single modality.

We also demonstrated reduction in the size of cystic follicles with increased thickness of granulosa cell layer in PCOS‐IR rats that received metformin or empagliflozin. Use of combined treatment of metformin and empagliflozin showed apparently normal growing follicles at different stages of maturation with disappearance of cystic follicles.

In accordance with our results, study done by Wu et al., 2018 investigating the combined effect of metformin and pioglitazone on the testosterone levels and follicular development in a rat model of PCOS found that metformin treatment significantly inhibited the level of free and total testosterone (TT). They also verified that metformin treatment improved follicular development in a rat model of PCOS, with reduced proportions of primary and cystic follicles and larger proportions of early antral follicles. Study done by Rafiee et al., 2022 revealed that metformin treatment of PCOS‐affected ovaries in mice, increased the number of corpus luteum, primordial and graafian follicles; while decreased the number of primary, secondary, atretic follicles, and ovarian cysts.

Many studies showed that analysis of follicular count in PCOS models showed significant increase in the numbers of preantral follicles, the numbers of atretic follicles, and ratios of atretic follicle to the total numbers of follicles, while the numbers of primordial follicle pool, antral follicles, and corpora leutea were significantly decreased as compared to the control groups (Wu et al., 2014; Furat et al., 2018). Accordingly, the sum of Graafian follicles, displaying a large fluid‐filled antral cavity surrounded by granulosa cells and containing oocyte, and the corpora leutea were estimated in our work.

Another randomized controlled trial discovered that a brief, 2‐day regimen of metformin; effectively reduced the testosterone levels caused by LH in PCOS women. They proposed that metformin has an immediate impact on androgen secretion and/or production at the ovarian level, separate from its effects on insulin sensitivity (Kurzthaler et al., 2014).

Previous studies have shown the beneficial impact of metformin on hormonal disturbances in PCOS; A study by Hu et al. (2020) on the impact of metformin on PCOS in rats revealed that metformin‐treated animals had significantly improved levels of LH, testosterone, FSH, and LH/FSH ratio compared to model rats. They also showed that the number of granulosa cells and the production of lutein were significantly improved in the metformin‐treated group, while cystic follicle enlargement was missing. Our findings are consistent with those of Zaheer et al., 2021, who examined the impact of canagliflozin and metformin on the estrous cycle and hormonal dysregulation of letrozole‐induced PCOS in rats and came to the conclusion that canagliflozin is effective alone and enhances the impact of metformin in the regulation of estrous cycles and reversal of hormonal disturbances in a rat model of PCOS. According to reports, canagliflozin enhances B‐cell activity by lowering insulin demand and hyperinsulinemia, which are major contributors to the rise in testosterone levels in PCOS. On the other hand, a meta‐analysis was carried out by Sinha and Ghosal (2022) to highlight the effect of SGLT‐2 inhibitors on the metabolic and hormonal aspects of PCOS. They discovered that, aside from the effect on dehydroepiandrosterone sulphate (DHEAS), there was no significant difference seen for the free androgen index (FAI), TT, and sex hormone binding globulin (SHBG).

The reduction in DHEAS demonstrated here raises an intriguing hypothesis whereby SGLT‐2 inhibitors, by virtue of their special capacity to lower body weight and enhance glucose uptake, would decrease hyperinsulinemia, along with a reduction in DHEAS leading to a reduction in free testosterone, which in turn would improve glucose utilization, forming a basis for breaking the vicious cycle of hyperinsulinemia and hyperandrogenism, which is the foundation of PCOS.

Uncertainty exists regarding the process through which IR advancements improve PCOS's endocrine and reproductive systems. In present study, we demonstrated that letrozole combined with a high fat diet for 27 days exhibited features of PCOS‐IR with significant decrease in the relative expression of AMPKα and SIRT‐1 in ovarian tissue. Besides, the expression levels of AMPKα and SIRT‐1 in ovarian tissue were upregulated in the PCOS‐IR rat model which received empagliflozin, metformin monotreatment and combined drug treatment of for 28 days.

Many studies have found that AMPK agonists, such as metformin, can improve PCOS endocrine and reproductive function (Fulghesu et al., 2012; Kiyak Caglayan et al., 2015), which coincides our experimental findings. Study done by Tao et al., 2019 demonstrated that 4 weeks treatment of PCOS group with metformin has shown a substantial improvement in body weight, fasting blood sugar, and HOMA‐IR with an increase in AMPK and SIRT1 expression compared to the PCOS group that had not received treatment. Another in vitro study in women with PCOS found that metformin could improve granulocyte function by reducing TNF‐ and chemokine‐mediated inflammatory responses (Kai et al., 2015). Also, it has been found that SIRT1 levels will be decreased in patients with PCOS (Kiyak Caglayan et al., 2015).

It has been established that SIRT1 and AMPK are closely related since activating AMPK significantly increases SIRT1 activity (Oliva et al., 2008). Coincidentally, a prior study discovered that the AMPK agonist metformin significantly raised SIRT1 levels and enhanced reproductive endocrine function in PCOS rats (Tao et al., 2015).

According to a study done by Lee et al. (2021), ipragliflozin treatment over long period of time has shown favorable metabolic effects on mice with HFD induced obesity. They reported that SGLT2 inhibitor boosted AMPK phosphorylation, increasing SIRT1 in the liver and WAT as a result. Canagliflozin has also been observed to increase adipocyte cellular energy expenditure by inducing mitochondrial biogenesis through the AMPK‐Sirt1‐Pgc‐1 signaling pathway (Yang et al., 2020).

Other in vitro study on HG‐treated human umbilical vein endothelial cells (HUVEC) revealed that HG results in inactivation of AMPK/SIRT1/PGC‐1α axis, whereas Dapagliflozin treatment restored SIRT1, PGC‐1α, and p‐AMPK (Faridvand et al., 2022).

PCOS is considered a pro‐inflammatory state where T2DM and cardiovascular illnesses are linked to this inflammatory condition (Vgontzas et al., 2003). According to previous reports, PCOS patients have higher expression of cytokines that promote inflammation (González, 2012; Zhang et al., 2014). Inflammation explains the link between the metabolic aspects of PCOS like insulin resistance, diabetes, obesity, and cardiovascular disease (Osborn & Olefsky, 2012).

Studies suggested that an imbalance of anti‐inflammatory and pro‐inflammatory cytokines may result in disturbed ovarian function, altered steroidogenesis, and diminished follicular maturation (Vural et al., 2010).

In the present study, use of metformin or empagliflozin showed significant improvement in inflammatory markers; TNF‐α and IL6 and use of combined treatments demonstrated superior significant anti‐inflammatory effect in PCOS‐IR rat model as compared to single modality. Numerous studies using human and animal cells, such as human umbilical vein endothelial cells or bovine aortic endothelial cells, have demonstrated that metformin reduces the inflammatory state caused by hyperglycemia by suppressing the nuclear factor kappa B (NF‐B) signaling pathway through AMPK‐dependent and independent pathways. Suppression of NF‐κB decreases the expression of pro‐inflammatory mediators like interleukin 6, interleukin 1β, and tumor necrosis factor alfa (TNFα). Metformin can also indirectly inhibit chronic inflammation through enhancing insulin sensitivity, regulating hyperglycemia, preventing formation of advanced glycation end‐products (AGEs), and diabetic atherogenic dyslipidemia (Kuryłowicz & Koźniewski, 2020; Nasri & Rafieian‐Kopaei, 2014; Zhou et al., 2018).

In agreement with our results, study done by Bonora et al., 2020 reported that SGLT‐2 inhibitors significantly improved serum levels of inflammatory markers like IL‐1β, IL6, and TNFα.

According to previous studies, SGLT‐2 inhibitors help to minimize oxidative stress and inflammatory reactions (Han et al., 2017; Leng et al., 2016). It has been demonstrated also that use of SGLT‐2 inhibitors can drastically reduce both the infiltration of macrophages and the expression of inflammatory M1 markers including TNF‐, IL‐1, and IL6 (McKellar et al., 2009; Moriya, 2019). The underlying molecular pathway of the anti‐inflammatory effects of SGLT‐2 inhibitors are still not fully understood. However, they might behave somewhat similarly to metformin. In vitro, it has been discovered that SGLT‐2 inhibitors activates AMPK (directly or by boosting adiponectin expression (Xu et al., 2017).

Obesity is one of the most common disorder that complicates the PCOS (Dumesic et al., 2015). The present study showed that empagliflozin, metformin monotreatment, and combined drug treatment of PCO‐IR rat model for 28 days exhibited similar significant improvement in the elevated BMI with no added significant effect for the combined treatment over single modality.

Our results are in line with previous studies, which showed that taking metformin can reduce BMI in PCOS patients (Li et al., 2020; Tan et al., 2008). The Diabetes Prevention Program reveals that the main weight‐loss mechanisms of metformin is through improving IR and reducing food intake (Yerevanian & Soukas, 2019). It has been shown that circadian rhythm and gastrointestinal physiology changes caused by metformin also help in controlling fat oxidation and storage in the liver, skeletal muscles, and adipose tissues (Bridgeman et al., 2018; Malin & Kashyap, 2014).

Furthermore, a growing body of evidence has shown that SGLT2 inhibitors decrease body and fat mass by enhancing fat oxidation and caloric loss through glycosuria. They can also activate lipolysis, and thus promote higher fat utilization, in obese animal models (Obata et al., 2016; Osataphan et al., 2019; Suzuki et al., 2014; Yokono et al., 2014). Xu and Osataphan have reported, FGF21 seems to mediate, at least in part, SGLT2 inhibitor‐induced activation of lipolysis in adipose tissue (Osataphan et al., 2019; Xu et al., 2017).

In this study, We observed that both metformin and empagliflozin can improve the endocrine and metabolic functioning in rats with PCOS‐IR via the AMPK‐SIRT1 pathway which may be the molecular basis for IR in PCOS and may potentially be a therapeutic target. Numerous studies have demonstrated the effectiveness and safety of metformin in improving metabolic outcomes in PCOS, despite the fact that it lacks a license for this use. SGLT‐2 inhibitors are also promising therapeutics that have been shown significant advantages in improving metabolic abnormalities in women with PCOS, however their roles and pathways in improving PCOS metabolic and hormonal disturbances need further studies. It is crucial to recognize that no single treatment can cover the whole metabolic disorders in women with PCOS, so, combination of more than single modality to improve the parameters of metabolic disorders is highly recommended.

## AUTHOR CONTRIBUTION

I declare that all data were generated in‐house and that no paper mill was used.

## FUNDING INFORMATION

Self‐funded.

## CONFLICT OF INTEREST STATEMENT

No competing interests for all authors.

## ETHICS STATEMENT

This study involves animals where the Institutional Ethics Committee's guidelines for the proper handling and use of laboratory animals were followed, as were all operations involving animals.

## INFORMED CONSENT

Not applicable.

## Data Availability

The data that support the findings of this study are available on request from the corresponding author. The data are not publicly available due to privacy, legal, or ethical restrictions. Data generated or analyzed during this study are provided in full within the published article.
